# Case Report: Anaphylactic shock induced by cefoperazone sodium and sulbactam sodium for injection

**DOI:** 10.3389/fmed.2025.1579601

**Published:** 2025-06-18

**Authors:** Weiqian Liu, Bo Gao, Shaowei Xie, Dongrui Yan, Yu Yin

**Affiliations:** ^1^Department of Rehabilitation Medicine, Hebei General Hospital, Shijiazhuang, China; ^2^Hebei Provincial Key Laboratory of Cerebral Networks and Cognitive Disorders, Hebei General Hospital, Shijiazhuang, China; ^3^Department of Urinary Surgery, Hebei General Hospital, Shijiazhuang, China

**Keywords:** case report, injectable cefoperazone-sulbactam, anaphylactic shock, adverse drug reactions, antibiotics

## Abstract

**Rationale:**

Cefoperazone-sulbactam is a widely used antibiotic in clinical practice, and anaphylactic shock is a severe adverse drug reaction. Previous literature has rarely reported cases of anaphylactic shock induced by cefoperazone-sulbactam.

**Patient concerns:**

A 38-year-old male patient who underwent surgery for cerebral hemorrhage developed fever, with considerations of pulmonary and urinary tract infections. Following the administration of injectable cefoperazone-sulbactam, he experienced anaphylactic shock.

**Diagnosis:**

Anaphylactic shock was suspected to be caused by injectable cefoperazone-sulbactam.

**Interventions:**

The patient was administered a subcutaneous injection of 1 mg epinephrine, followed by continuous infusion of 10 mg norepinephrine in 45 mL of 5% glucose solution via a pump. Additionally, assisted ventilation with a respirator and fluid resuscitation were provided.

**Outcome:**

The patient was discharged with improved symptoms.

**Lessons:**

To prevent serious adverse reactions, it is essential to strictly adhere to indications and contraindications to avoid misuse and minimize the occurrence of adverse effects. Prior to medication administration, a thorough inquiry into the patient’s drug allergy history and family history should be conducted.

## 1 Introduction

The injectable formulation of cefoperazone sodium and sulbactam sodium is a compound preparation consisting of sterile powder that uniformly mixes cefoperazone sodium and sulbactam sodium. Cefoperazone sodium is a third-generation cephalosporin antibiotic, which exerts its bactericidal effect primarily by inhibiting the synthesis of bacterial cell walls. Sulbactam sodium, as a β-lactamase inhibitor, protects cefoperazone sodium from hydrolysis by β-lactamases, thereby enhancing the efficacy of cefoperazone sodium. This medication is mainly indicated for the treatment of infections caused by susceptible bacteria in the respiratory system, urinary system, reproductive system, and intra-abdominal infections (1, 2). The sodium cefoperazone and sulbactam combination exhibits advantages such as a broad antibacterial spectrum, strong antimicrobial efficacy, and low toxicity, leading to its widespread clinical application in recent years. Adverse reactions associated with this medication include allergic responses, which may manifest as maculopapular rashes, urticaria, eosinophilia, and drug fever (3). In rare cases, patients may experience severe adverse effects (4). This report presents a case of anaphylactic shock induced by the injection of sodium cefoperazone and sulbactam.

## 2 Case presentation

The patient is a 38-year-old male who was admitted due to limited mobility in the limbs accompanied by cognitive impairment for over 4 months. On October 8, 2024, he was diagnosed with the following conditions: (1) Limb paralysis. (2) Cognitive impairment. (3) Recovery phase of cerebral hemorrhage. (4) Stage 3 hypertension (very high risk). (5) Post-tracheostomy status. (6) Pulmonary infection. (7) Post-arterial duct occlusion procedure. (8) Mild anemia. (9) Hypoproteinemia. (10) Urinary tract infection. The patient’s condition is stable, and he is undergoing regular rehabilitation therapy.

The patient exhibited fever upon waking on the morning of November 2, 2024, with a recorded temperature of 38.2°C. Symptomatic treatment was initiated with L-lysine acetylsalicylate for antipyretic purposes, and family members were advised to implement physical cooling measures and ensure adequate hydration. Subsequently, the patient’s body temperature continued to rise, accompanied by chills. Blood cultures were collected for analysis. Around 11:50 AM, dexamethasone sodium phosphate injection was administered as an adjunctive therapy for inflammation and fever reduction; however, the patient’s temperature remained elevated, reaching between 39°C–40°C. Laboratory Results Report: Emergency Blood Cell Analysis + C-Reactive Protein: C-Reactive Protein: 47.02 mg/L ↑; White Blood Cell Count (WBC): 16.11 × 10^9^/L ↑; Neutrophils (NEUT): 78.90% ↑; Lymphocytes (LYMPH): 13.00% ↓; Neutrophils (NEUT): 12.70 × 10^9^/L ↑; Procalcitonin PCT (Fluorescent Quantitative Method): <0.04 ng/mL; Urinalysis + Urinary Formed Elements Analysis: Hematuria: 3 + Abnormal; Proteinuria: 1 + Abnormal; Nitrite: + Abnormal; Leukocytes: 3 + Abnormal; Red Blood Cells: 3216.5/μL ↑; Red Blood Cells/HPF: 578.97 cells/HPF ↑; Leukocytes: 782.1/μL ↑; Leukocytes/HPF: 140.78 cells/HPF ↑; Bacteria: 17316.9/μL ↑; Bacteria/HPF: 3117.04 cells/HPF ↑. Considering the possibility of pulmonary and urinary tract infections in the patient, symptomatic anti-infective treatment was administered around 12:30 with cefoperazone sodium and sulbactam sodium (Shu Pu Shen, Pfizer Pharmaceutical Co., Ltd.). Due to the unavailability of ice blankets, patients were provided with physical cooling through saline solution and fluid replacement therapy. At 14:50, the patient presented with a body temperature of 40 degrees Celsius and new-onset generalized skin rashes. The patient was administered intramuscular diphenhydramine and received nebulization therapy. The patient experienced a sudden drop in blood pressure, with electrocardiographic monitoring indicating a heart rate of 150–160 beats per minute, systolic blood pressure ranging from 50 to 60 mmHg, and an undetectable pulse oximetry reading. Physical examination revealed weak pulsations in the major arteries, and the patient’s vital signs were unstable. An urgent consultation with the intensive care unit (ICU) was requested. Impression: Allergic shock? Infectious shock? The patient was administered 1 mg of epinephrine via subcutaneous injection, along with a continuous infusion of norepinephrine at a dosage of 10 mg mixed in 45 mL of 5% glucose solution. Additionally, mechanical ventilation support and fluid resuscitation therapy were initiated. The patient’s blood pressure increased to approximately 90/50 mmHg. Suggestion 1: Continue the infusion of vasopressor agents to elevate blood pressure, while considering the possibility of anaphylactic shock. It is recommended to discontinue any suspected allergenic medications. If conditions permit, administer a solution of 5% glucose (49 mL) combined with epinephrine (1 mg) via infusion. Suggestion 2: The possibility of septic shock should not be excluded; therefore, continue with anti-infective therapy and fluid resuscitation. Suggestion 3: Monitor changes in blood pressure and heart rate closely. Our department will provide follow-up care. The patient was given fluid resuscitation and vasopressor treatment, along with physical cooling using an ice blanket. Invasive mechanical ventilation was employed to assist in improving the patient’s respiratory status. Subsequently, the patient’s blood pressure stabilized, prompting adjustments to the norepinephrine infusion rate based on blood pressure readings. Meropenem was administered for anti-infective treatment as well. The patient was later transferred to the ICU for further management and subsequently showed improvement before being discharged from the hospital.

## 3 Discussion

The issue of whether a skin allergy test is necessary prior to the use of cephalosporin antibiotics has long been a topic of debate. In China, various descriptions can be found in drug instructions and reference materials; however, both the 2010 edition of “Clinical Medication Guidelines” and the 2015 edition of “Guidelines for Clinical Application of Antibacterial Drugs” do not mandate skin allergy testing for cephalosporins. According to the consensus reached at the “High-End Forum on Skin Allergy Testing for Cephalosporin Antibiotics,” it is agreed that if the drug instructions explicitly state that a skin allergy test must be conducted before use, then such testing is obligatory. Conversely, if there is no clear stipulation in the drug instructions, clinicians should comprehensively consider factors such as whether the patient has an allergic constitution, any history of previous drug allergies, and the severity of their condition when deciding whether to perform a skin allergy test (5). According to the Naranjo scale (Table 1), which assesses the probability of adverse reactions related to medications, this algorithm received a score of 5, indicating a high likelihood of correlation. Previous analyses of adverse drug reactions (ADRs) associated with cefoperazone sodium/sulbactam sodium in domestic studies have indicated that the most common manifestations involve skin and its appendages, as well as systemic damage. The primary clinical presentations include rash, pruritus, erythema, allergic reactions, and anaphylactic shock (6, 7). Liu and Fu (8) conducted a study that revealed among 109 cases of adverse drug reactions (ADRs) related to cefoperazone/sulbactam in the Beijing area, there were 51 instances of skin and appendage damage and 9 instances of systemic reactions, accounting for a total of 55.05%. Similarly, Li et al. (9) found that out of 518 cases associated with cefoperazone/sulbactam-induced ADRs, there were 164 occurrences of skin and appendage damage and 160 occurrences of systemic damage, together constituting 62.55%.

**Table 1:** Naranjo ADR evaluation scale.

	Score		
Related questions	Yes	No	Unknown
1. Is there any conclusive report before this ADR?	1√	0	0
2. Do the ADR occur after the use of suspect drugs?	2√	−1	0
3. Do the ADR relieve after drug withdrawal or use of antagonist?	1√	0	0
4. Does the ADR recur after reuse of the suspect drug?	2	−1	0
5. Are there other reasons that can cause the ADR independently?	−1	2	0
6. Does the ADR repeat after the application of placebo?	−1	1	0
7. Does the drug reach toxic concentration in blood or other body fluids?	1	0	0
8. Is the ADR aggravated (relieved) with the increase (decrease) of dose?	1√	0	0
9. Has the patient ever been exposed to the same or similar drugs and had similar reactions	1	0	0
10. Is there any objective evidence to confirm the reaction?	1	0	0
Total score	5		

The total score ≥9 shows that the causal relationship of adverse drug reactions is definite; the total score 5–8 is probably or likely to be relevant; the total score 1–4 is possible to be relevant; the total score ≤0 is doubtful to be relevant. ADR, adverse drug reactions.

The occurrence of severe allergic reactions is closely related to the intrinsic allergens present in the medications used and the individual patient’s allergic constitution. Cephalosporins themselves are non-immunogenic; rather, it is their high molecular weight polymer impurities that serve as the primary allergens (10). Lin (11) conducted an analysis of 328 cases of allergic shock induced by cephalosporin antibiotics, indicating that a history of allergies is a significant factor contributing to the occurrence of allergic shock associated with this class of drugs. Furthermore, the incidence of allergic shock was found to be unrelated to gender or age. The findings reported in the literature (12) suggest that the peak period for allergic shock following administration occurs within 30 min after receiving cefoperazone/sulbactam, which aligns with our case. In the event of an allergic reaction, it is imperative that the medication be discontinued immediately and appropriate management measures be implemented.

The following suggestions are made regarding the clinical use of antibiotics such as penicillin and cephalosporins, which are known to potentially induce allergic reactions. It is essential to strictly adhere to the indications and contraindications for these medications in order to prevent misuse and minimize the occurrence of adverse reactions (13). Prior to administration, a thorough inquiry into the patient’s history of drug allergies and family history should be conducted. These antibiotics are contraindicated in individuals with known hypersensitivity to penicillins, sulbactam, or cephalosporin-class antibacterial agents. Post-administration monitoring should be intensified, particularly for elderly patients and those with a predisposition to allergies.

## Data Availability

The original contributions presented in the study are included in the article/Supplementary material, further inquiries can be directed to the corresponding author.
